# The Impact of the US Priority Review Voucher on Private-Sector Investment in Global Health Research and Development

**DOI:** 10.1371/journal.pntd.0001750

**Published:** 2012-08-28

**Authors:** Andrew S. Robertson, Rianna Stefanakis, Don Joseph, Melinda Moree

**Affiliations:** BIO Ventures for Global Health, San Francisco, California, United States of America; McGill University, Canada

Diseases such as trypanosomiasis, leishmaniasis, and lymphatic filariasis impose substantial health burdens in developing countries [1], [2]. These diseases are widely neglected because there is little financial incentive for biopharmaceutical companies to invest in developing new treatments, vaccines, and diagnostics. Most of the 1,000,000,000 people affected by neglected diseases are poor and live in low-income countries [3]. Although there is a significant need for new cost-effective drugs and vaccines, from 1975–1999 less than 1%–2% of new chemical entities marketed were for tropical diseases and tuberculosis [1], [4].

Market incentives play an important role in mobilizing companies toward neglected tropical disease (NTD) research. Pharmaceutical companies have a fiduciary responsibility to shareholders to maximize profits. Absent of other incentives, companies will focus research and development (R&D) on products and programs that possess a profitable market, have a sufficient likelihood of technical success, and are likely to achieve a maximum return on investment. In contrast, the global burden of neglected infectious diseases is concentrated in developing countries with inadequate health budgets and poor patients who can pay only low prices for drugs, if they can afford to pay at all. Drugs and vaccines that target neglected diseases thus typically cannot compete with potentially profitable products for internal company or investor R&D dollars.

Governments and foundations have recognized the dearth of private-sector incentives for investment in NTD research and have responded with “push” funding (up-front funding for drug development) and “pull” mechanisms (rewards for output) to promote successful development [5], [6]. The priority review voucher (PRV) program, currently administered by the US Food and Drug Administration (FDA), was passed into United States law in 2007 as a pull mechanism to help promote R&D for new medicines targeting NTDs, malaria, and tuberculosis [7]. Under this law, companies that receive FDA approval for a novel drug or vaccine targeting one of 16 tropical diseases are awarded a transferable voucher. This voucher can be sold to a second organization or can be redeemed to grant the bearer priority six-month review for a future medicine of their choosing [8]. As average standard review periods can range between 10–16 months, the voucher could potentially allow drugs to reach the market up to eight months earlier. Economic models have predicted that this faster time to market could be worth between US$50 million to US$300 million [9], [10].

However, the impact of the PRV incentive in developing new medicines for NTDs has been questioned, in part due to uncertainty around the value of the voucher among pharmaceutical and biotech companies [11], [12]. The PRV program as implemented carries restrictions that make earning or using a voucher difficult or impractical, such as a requirement that developers provide notice to the FDA at least one year prior to the use of a voucher (often before clinical trials have concluded). Further, the voucher has not resulted in demonstrated value for any company to date. At writing, Novartis is the only company to have received a PRV, which they used in 2011 to accelerate the review of a supplemental new drug application (sNDA) for their gouty arthritis drug candidate Ilaris (canakinumab) [13]. The FDA fulfilled their responsibilities under the PRV program to conduct a six-month priority review, but ultimately denied approval of Ilaris citing the need for further data to assess the overall safety profile [14]. It is possible that Novartis gained some benefit from the use of the PRV for the review of Ilaris, but this value is difficult to quantify.

## Understanding the Influence of the PRV

Despite the uncertainty surrounding its value, the PRV has had some influence on the private sector. From March to July 2011, BIO Ventures for Global Health conducted a survey of companies with active drug or vaccine programs in one of the 16 PRV-eligible diseases. The purpose of the survey was to investigate 1) the influence of the PRV in initiating and continuing NTD product development, 2) the perceived monetary value of the PRV, and 3) improvements needed for the PRV program. Brief online surveys with ten questions were electronically distributed to executives of 24 for-profit companies pursuing active R&D of a drug or vaccine that would likely receive a PRV upon approval [15] (Text S1). We received responses from 12 companies representing 27 unique NTD drug or vaccine programs: seven responses from “small” companies with fewer than 60 full-time employees (FTEs) and five responses from “large” companies with over 500 FTEs. Quantitative and qualitative survey results were aggregated and anonymized. All respondents were invited to participate in a follow-up interview to verify and better clarify the results of the survey; seven respondents—six small companies and one large company—agreed.

**Figure 1:**
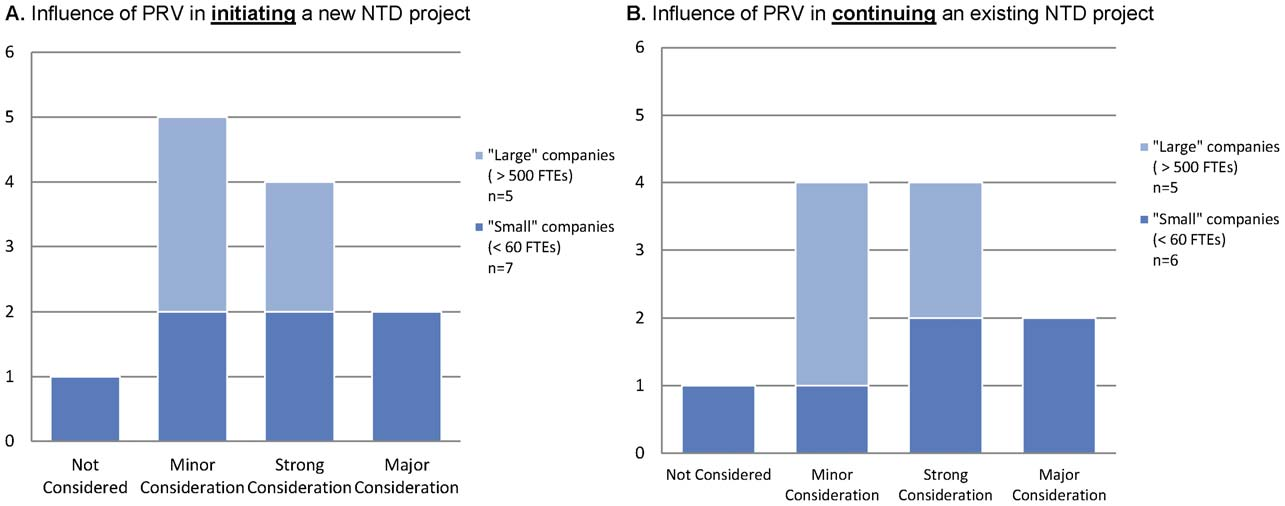
Industry response to priority review voucher (PRV) influence on decision making. Companies rated the importance of the PRV on initiating (A) and continuing (B) R&D projects for new neglected tropical disease (NTD) medicines. Responses were identified into “large” companies (>500 FTEs) and “small” companies (<60 FTEs). Note: One small company did not respond to the survey question that supplied the data for (B).

Ten of the 12 respondents indicated they were “somewhat” or “very” familiar with the PRV program, and two respondents indicated they were “aware but unfamiliar” with the PRV program. Respondents were asked to indicate the level of consideration given to six identified incentives commonly referenced in neglected disease research, including the PRV, with possible responses listed in context and comprising of “not considered,” “minor consideration,” “strong consideration,” and “major consideration.” The majority of companies (91% of respondents) indicated that the PRV was a “strong” or “major” consideration of their organization during the process of initiating or continuing their company's respective neglected disease project (Figure 1) (note that one company was nonresponsive to the question regarding the influence of the PRV in continuing their company's NTD project). Of these, six companies (50% of respondents) indicated that the voucher was a “strong” or “major” factor in deciding whether to pursue a particular development project. Further, follow-up interviews with two “small” company respondents indicated that the voucher is a necessary incentive for the development of their respective NTD programs.

When compared against other possible motivating factors, however, responses suggest that other factors were of more significance than the PRV when considering the creation and continuation of a neglected disease vaccine or drug (Table 1). Responses for the importance of the six individual commonly-referenced factors were weighted on a scale of 0 to 3 (“not considered” = 0; “minor consideration” = 1; “strong consideration” = 2; “major consideration” = 3), and comparative rankings were created based on the cumulative score for each factor.

**Table 1:** Ranked considerations for initiating and continuing a neglected tropical disease (NTD) project.

	(A) Ranked considerations for initiating an NTD project			(B) Ranked considerations for continuing an NTD project		
Rank	All companies(n = 12)	“small” companies(<60 FTEs, n = 7)	“large” companies(>500 FTEs, n = 5)	All companies(n = 11)	“small” companies(<60 FTEs, n = 6)	“large” companies(>500 FTEs, n = 5)
**1**	Developing world or emerging markets	Developing world or emerging markets	Employee morale	Developing world or emerging markets	Developing world or emerging markets	Employee morale
**2**	Goodwill and corporate social responsibility	Developed world markets	Goodwill and corporate social responsibility	Developed world markets	Developed world markets	Goodwill and corporate social responsibility
**3**	Non-market-based incentives	Non-market-based incentives/**Priority review voucher**	Developing world or emerging markets/Non-market-based incentives	Non-market-based incentives/Employee morale	Non-market-based incentives/**Priority review voucher**	Non-market-based incentives/Developing world or emerging markets
**4**	**Priority review voucher**	Goodwill and corporate social responsibility	**Priority review voucher**	Goodwill and corporate social responsibility	Goodwill and corporate social responsibility	Developed world markets
**5**	Employee morale/Developed world markets	Employee morale	Developed world markets	**Priority review voucher**	Employee morale	**Priority review voucher**

Potential market value in “developing world or emerging markets” was the top-ranked reason for initiating and continuing an NTD project. Responses for the importance of the six individual, commonly referenced factors were weighted on a scale of 0 to 3 (“not considered” = 0; “minor consideration” = 1; “strong consideration” = 2; “major consideration” = 3), and comparative rankings were created based on cumulative scores for each factor. Rankings are also broken down into “large” companies (>500 FTEs) and “small” companies (<60 FTEs). The number (n) of companies that responded is also indicated; one small company did not respond to the survey question that supplied the data for (B).

Overall, the PRV ranked fourth and fifth as a consideration for initiating and continuing an NTD program, respectively. By contrast, “potential market value in the developing world or emerging markets” ranked as the most significant influencing factor when starting or continuing a NTD project, with eight companies (67%) listing potential markets as a “strong” or “major” consideration when initiating an NTD project. Other factors, such as “good will and corporate social responsibility” and “non-market-based incentives (such as contracts and grants)”, also rated higher than the PRV. Further, smaller companies considered the PRV as a higher relative priority than did larger companies.

Follow-up interviews helped resolve the apparent disparity between the high individual influence and the low relative importance that the PRV has in prioritizing R&D projects. All seven respondents interviewed indicated that the novelty of the voucher program, including the lack of a demonstrated sale of a voucher by one organization to a second organization, makes the value of the voucher difficult to determine. Rather, the PRV's influence on research priorities is “part of a larger conversation concerning neglected diseases.” In many instances, the PRV has served as “a carrot to help engage investors,” and has assisted companies to “initiate serious thinking about neglected disease programs, which often go undiscussed.” However, with the exception of two NTD programs, the PRV was never cited as an independently sufficient motivating factor for initiating or continuing an NTD project. Instead, the PRV was viewed as an additional incentive for pursuing an NTD project, but needed to be coupled with incentives such as nondilutive funding or advanced purchase agreements in order to have a significant effect on a company's strategy.

## Understanding the Value of the PRV

In addition to understanding the impact that the PRV has had on portfolio prioritization, we sought to gain insight into the monetary value of the voucher as seen by the for-profit sector. To date, the value of the PRV has only been described through economic models and has ranged from between US$50 million to US$300 million. However, the “value” of a commodity (such as a voucher) is difficult to determine with any precision and is highly relative. For example, the amount of benefit that a company may get from using a voucher will likely be different from the amount of money that a company may spend to obtain a voucher.

We sought to investigate two indicators that would help describe the financial value of the PRV as an incentive. The first indicator, “pre-market value,” was defined as the additional investment that companies are willing to make to obtain a PRV: that is, the additional amount companies would spend on drug development if they would receive a voucher in return for FDA approval. The second indicator, “expected market value,” was defined as the amount companies would expect to receive for the sale of a PRV to another organization. The “pre-market value” indicator reflects the increased amount the voucher-seeking company will invest in global health R&D, while the “expected market value” reflects the perceived benefit a PRV would bring to a company that pursues a voucher.

**Figure 2:**
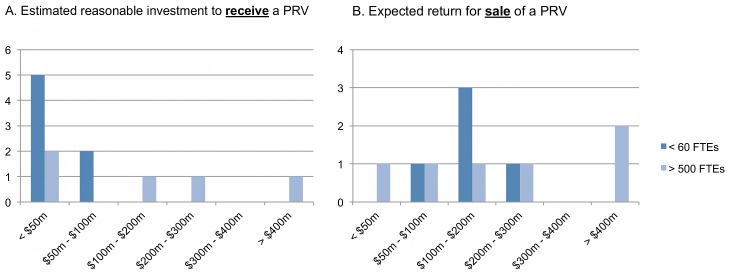
Industry perspectives with regard to priority review voucher (PRV) value. (A) Companies were asked to approximate the amount their company would spend to earn a PRV. On average, respondents indicated their company would find reasonable spending US$94 million (mode = US$25 million; median = US$25 million; SD = ±US$119 million). (B) Respondents were asked to approximate the amount their company would expect to receive for the sale of a PRV. On average, respondents indicated their company would expect to receive US$188 million (mode = US$150 million; median = US$150 million; SD = ±US$142 million). Responses were identified into “large” companies (>500 FTEs) and “small” companies (<60 FTEs).

There was considerable variation among respondents on the pre-market value of the PRV. On average, respondents were willing to spend US$94 million to obtain a PRV (Figure 2A). However, the individual responses varied significantly, with a standard deviation (SD) value of ±$119 million. Responses received from the seven “small” companies (<60 FTEs) indicated a willingness to spend an average of US$39.3 million to obtain a PRV and a SD value of ±US$22.5 million. Responses received from the five “large” companies (>500 FTEs) indicated a willingness to spend an average of US$170 million to obtain a voucher with a significant SD of ±US$142.7 million.

The expected market value estimates were higher than pre-market value estimates and suggested that companies expect about a twofold return on investment for a PRV. On average, respondents expected to receive US$179.6 million for the voucher (Figure 2B). Again however, the responses varied significantly, with a SD value of ±US$123.4 million. Predicted market value by small companies was more consolidated, averaging at US$154.2 million (SD = ±US$50.8 million). Correspondingly, large companies had a more varied prediction of market value for the voucher, averaging US$216.7 million (SD = ±US$147 million).

The wide range of responses regarding the value of the voucher suggests that there is no industry consensus on the value of the PRV. While small companies came closer to a consensus regarding the pre-market value, follow-up interviews suggest that this may reflect capital constraints that smaller companies face; given greater access to early development capital, the pre-market value of the voucher to smaller companies may increase. Regardless, these results suggest that there is some recognized value of the PRV; however, the industry perception of the voucher has not normalized and is still clouded by significant uncertainty.

## Improving the PRV as an Incentive

While the PRV holds some incentive value to companies, there are steps that can be taken to improve this value as an incentive. During follow-up interviews, we asked respondents to identify possible changes or events that, if realized, would improve the PRV as an incentive. First and foremost, respondents overwhelmingly stressed the need for a demonstrated sale of a voucher, with the purchase price disclosed to developers. While not definitive in establishing the market value of the voucher, respondents indicated that this event would “provide concrete input from large companies as to what the voucher is worth,” would help build credibility around the program, and would give the PRV a “sticker price.” Without a better understanding of the actual market for a voucher, the PRV's value is uncertain and difficult to assess.

Respondents also underscored the need for the FDA to demonstrate support for the PRV program. While this was not a universal priority, developers often discussed “general cynicism for the FDA,” and uncertainty as to the FDA's intent to fulfill their obligations under the program. Developers cited the voucher user fee (currently set to US$5.2 million) [16] as demonstrating a lack of support from the FDA for the voucher program. However, it should be noted that this survey was conducted prior to the FDA review of Novartis's Ilaris and may have changed in subsequent months, given that the FDA gave a decision on Ilaris within the six-month priority review timeline. Also, the legislation requires the FDA to set the fee based on the average cost incurred by the agency during the review of a human drug application subject to priority review in the previous fiscal year [7].

Finally, developers cited the need for revision of implementation rules governing the PRV program. These revisions varied between respondents and included relaxing the requirement that companies issue a one-year advanced notice prior to redeeming a PRV, relaxing the limit on voucher transferability (currently restricted to one transfer of ownership per voucher), and providing greater clarity into the revision process for the list of PRV-eligible diseases. Steps recommended by respondents worked to either improve the flexibility that companies would have in pursuing a PRV or increase the transparency regarding the issuance or use of a voucher.

Interestingly, when interviewed respondents had mixed opinions regarding what activities should be eligible for receipt of a PRV. This point has been debated previously in several contexts, including the award of a PRV to Novartis for Coartem despite its having already been available for a decade and registered in 85 countries [12], [17] and the proposed expansion of the list of PRV-eligible diseases to include rare pediatric diseases through the Creating Hope Act of 2011 [18]. Companies in favor of expanding the program to include diagnostics and additional diseases, and of relaxing the novelty requirement so that combination therapies would be eligible, had the perspective that this would increase opportunities to earn a voucher, thereby increasing the likelihood for a market valuation through a voucher sale. By contrast, companies that opposed expansion of the program did so with the view that increased opportunities to earn a voucher would dilute the voucher's value, thereby decreasing any realized return on investment.

While respondents were able to identify events that would improve the PRV as an incentive, they were hesitant to speculate on how these events would impact the monetary value of the voucher. This is understandable, as any change in value would be contingent on how the events unfolded. For example, a voucher may be resold for US$100 million or US$200 million, which in turn would have a different effect on the perceived value of a PRV.

## The Future of the PRV

Recent and pending events will likely impact the perception of the PRV. First and foremost is the impact that Novartis's use of their PRV will have on industry. Novartis earned a PRV in 2009 for their antimalarial drug Coartem. In June 2011, Novartis announced that they had used the voucher to gain priority review for Ilaris, a previously approved drug targeting gouty arthritis [13]. Although Ilaris was eventually not approved due to safety concerns, the FDA issued their decision within six months of the initial submission [14]. While this is only a single example, it could reassure companies considering the voucher that the FDA intends to fulfill their commitments under the PRV program. In this respect, it is also important to remember that the PRV program provides an accelerated process, and not a guaranteed outcome.

In addition, analysis of the drug development pipeline shows multiple voucher-eligible drugs currently in late-stage development. Current estimates suggest five vaccines and three drugs targeting a PRV disease are in phase III clinical trials alone [15]. Issuance of a voucher for any of these medicines, along with its subsequent sale, would provide a real-world example of how the voucher could be leveraged to provide a return on investment. Establishing a precise market value would require multiple sales of vouchers, providing several data points so that companies could make reliable predictions as to the potential return. While a single sale of a voucher would likely not provide enough information for companies regarding value, it would resolve much of the uncertainty surrounding the voucher's value to developers.

Further, the rules and regulations concerning the PRV program are evolving. The Creating Hope Act of 2011, currently under consideration in both the US Senate and House of Representatives, would change many of the current restrictions of the program [18]. These changes concern eliminating the one-time limitation on voucher transferability, reducing the one-year advanced notice required before voucher use, and clarification as to which drugs would be eligible for a voucher. In addition, the FDA is in the process of finalizing its draft guidance for the PRV, potentially clarifying many of the ambiguities of the voucher program [8]. Development of rules surrounding the voucher program could improve it as an incentive, reduce uncertainty, and increase its value to drug and vaccine developers.

## Rethinking Success

Our study begs reconsideration for what constitutes “success” when reviewing the PRV program. Initial expectations of the PRV program suggested that the voucher would serve as a broad-based incentive, and would influence companies to divert resources towards the development of new drugs and vaccines for neglected diseases [19]. Recent reviews of the PRV program have indicated that the voucher has failed in this respect, and is being overlooked by the for-profit sector. Our results demonstrate that the reality is more complex.

First, it is important to remember that the PRV program is designed as a cost-neutral incentive. The incentive program takes advantage of an inherent inefficiency in the drug and vaccine approval process—specifically, the fact that the FDA is overburdened and averages a 12–16 month period for standard drug review. One premise of the PRV program is that any costs incurred by the FDA would be offset by the redeeming company through the PRV user fee. Contrast this with incentive strategies that require direct funding, such as grant programs, advanced purchase agreements, or prizes. Under a cost-neutral approach such as the PRV program, *any* influx of funding or dedication of resources could be considered a “success.” From our interviews with developers, seven companies consider the PRV a positive incentive, and two companies cited the voucher as a necessary incentive to continue pursuing their particular neglected disease drug or vaccine R&D.

Second, the PRV should be viewed as an additional incentive rather than a “silver bullet.” As with all incentive programs, the PRV is just one consideration that would help companies decide whether or not to pursue a particular product or market. This is the same for other innovation incentives, including grants, contracts, and tax incentives, which cumulatively may tip the scale to make the development of a product favorable. Fifty percent of companies we surveyed indicated that the voucher was a significant factor in prioritizing product development, and our interviews confirm that most companies made portfolio decisions by taking all incentives—including the PRV—into account. Although the pre-market and expected market values of the PRV may not be enough to fully cover the costs of drug development, they help justify the investment alongside other incentives.

Finally, we must remember that the drug and vaccine product development process is long. Average development times from preclinical stages through submission of a new drug application are in the range of 10–20 years. Despite the fact that only one voucher has been issued to date, our survey results show that the PRV has either directly caused or influenced the initiation of product development targeting neglected tropical diseases. Looking prospectively, the demonstrated sale of a voucher from one company to another, or the reform of the PRV regulations, could increase the value of the voucher significantly. Given the apparent interest from industry working in the neglected disease space, these changes may provide a tipping point that could greatly increase the value of this R&D incentive.

## Supporting Information

Text S1 **Online survey instrument.** Electronically distributed survey to executives of 24 for-profit companies pursuing active R&D of a drug or vaccine that would likely receive a PRV upon approval.
(DOC)
