# Supplementary material for: The Impact of the US Priority Review Voucher on Private-Sector Investment in Global Health Research and Development
Source: PLoS Negl Trop Dis. 2012 Aug 28;6(8):e1750. doi: 10.1371/journal.pntd.0001750 (PMC3429395; doi:10.1371/journal.pntd.0001750)
Supplement: Text S1 — Online survey instrument. Electronically distributed survey to executives of 24 for-profit companies pursuing active R&D of a drug or vaccine that would likely receive a PRV upon approval. (DOC) [file pntd.0001750.s001.doc]

**Text S1. Online survey instrument.**

x

x
